# Chronic Respiratory Infection in Patients with Chronic Obstructive Pulmonary Disease: What Is the Role of Antibiotics?

**DOI:** 10.3390/ijms18071344

**Published:** 2017-06-23

**Authors:** Marc Miravitlles, Antonio Anzueto

**Affiliations:** 1 Pneumology Department, Hospital Universitari Vall d’Hebron, Ciber de Enfermedades Respiratorias (CIBERES), 08035 Barcelona, Spain; mmiravitlles@vhebron.net; 2 Department of Medicine, Division of Pulmonary Diseases/Critical Care Medicine, The University of Texas Health Science Center at San Antonio, San Antonio, TX 78229, USA; 3 Pulmonary Section, The South Texas Veterans Health Care System, Audie L. Murphy Memorial Veterans Hospital Division, Pulmonary Diseases Section (111E), 7400 Merton Minter Boulevard, San Antonio, TX 78229, USA

**Keywords:** chronic respiratory infections in COPD, exacerbations of chronic obstructive pulmonary disease, antibiotics, bacteria, prevention, colonization

## Abstract

Chronic infections are associated with exacerbation in patients with chronic obstructive pulmonary disease (COPD). The major objective of the management of these patients is the prevention and effective treatment of exacerbations. Patients that have increased sputum production, associated with purulence and worsening shortness of breath, are the ones that will benefit from antibiotic therapy. It is important to give the appropriate antibiotic therapy to prevent treatment failure, relapse, and the emergence of resistant pathogens. In some patients, systemic corticosteroids are also indicated to improve symptoms. In order to identify which patients are more likely to benefit from these therapies, clinical guidelines recommend stratifying patients based on their risk factor associated with poor outcome or recurrence. It has been identified that patients with more severe disease, recurrent infection and presence of purulent sputum are the ones that will be more likely to benefit from this therapy. Another approach related to disease prevention could be the use of prophylactic antibiotics during steady state condition. Some studies have evaluated the continuous or the intermittent use of antibiotics in order to prevent exacerbations. Due to increased bacterial resistance to antibiotics and the presence of side effects, several antibiotics have been developed to be nebulized for both treatment and prevention of acute exacerbations. There is a need to design long-term studies to evaluate these interventions in the natural history of the disease. The purpose of this publication is to review our understanding of the role of bacterial infection in patients with COPD exacerbation, the role of antibiotics, and future interventions.

## 1. Introduction

It is important to determine the role of bacteria and other pathogens in chronic obstructive pulmonary disease (COPD) patients with stable disease and during exacerbations. In these COPD patients, the isolation of “potentially pathogenic microorganisms” (PPMs) in respiratory samples ranges between 20% and 60% of cases [1,2,3]. The most common PPMs seen in COPD patients are *Hemophilus influenza*, *Moraxella catharralis*, *Streptococcus pneumonia*, *Pseudomonas* etc. [1,2,3]. The bacterial infection is predominantly found in the lower airway of these patient but can also be responsible for upper airway infections such as acute sinusitis. Some studies have suggested that these bacteria contribute to chronic airway inflammation leading to COPD progression [1,2,4]. Therefore, it has been suggested that the term chronic bronchial infection would be more appropriate when addressing the presence of significant concentrations of PPMs in the lower airways of stable COPD patients [2,5]. Patients with chronic bronchial infection may constitute a subgroup of individuals that may be called “infective phenotype” [2]. Our ability to identify bacteria by analysis of conserved 16S rRNA in bacteria has allowed the identification of the lung microbiota (present in the upper airway, sinus, bronchial tree etc.) [6]. We now recognize that the human’s airway is covered by a large variety of bacterial species that we were not able to culture using conventional methods [6]. The number of studies examining the microbiome of the lower airways is limited and there is some overlap between bacteria seen in COPD and healthy individuals [7]; however, a recent study has reported a significantly different bacterial community in patients with very severe COPD compared with nonsmokers, smokers and patients with cystic fibrosis [8]. Studies are clearly needed to understand the role of these microbiomes in healthy individuals and COPD patients and how to recognize that an “acute infection” is present; furthermore, we need to understand the impact of antibiotics—given for either acute exacerbations, or chronic long-term administration—on these bacterial communities.

The use of antibiotics in chronically infected patients may be associated with a reduction of bacterial load, and prevention of acquisition of a new bacterial strain; all these effects are associated with a reduction in the frequency and severity of COPD exacerbations. The role of prophylactic antibiotics for the prevention of COPD exacerbations was first studied during the 1950s and 1960s. The problem with these studies was that, at the time, we did not have an adequate definition of COPD; we had a small number of patients; we used narrow-spectrum antibiotics, and not well-defined end-points. After completion of these studies, there was increased concern regarding the development of bacterial resistance; therefore, no new studies were conducted for several years [9]. It was not until the late 1990s, with the availability of new classes of antibiotics and better understanding on the pathophysiology of COPD exacerbation, that new long-term antibiotic studies were conducted.

The most common causes of COPD exacerbations (ECOPD) are infections that are produced by bacteria (40–60%), viruses (about 30%) and atypical bacteria (5–10%) [10,11]. For the last 25 years, the clinical criteria described by Anthonisen et al. [12] have been incorporated in clinical guidelines to help in selecting patients that require empiric antibiotic therapy [13]. More recent studies have identified a change in color, for example, purulence is a good surrogate marker for the presence of bacterial infection [14,15,16]. Furthermore, only a change in sputum color was identified as a predictor of good response to antibiotics in a placebo-controlled clinical trial in patients with mild to moderate COPD [17]. Therefore, change in sputum color or increased purulence are the only clinical features that help clinicians to decide whether to use an antibiotic in ambulatory ECOPD. The purpose of this publication is to review the role of bacterial infection in patients with COPD both in stable conditions and exacerbation, as well as the role of antibiotics, and what other interventions can impact patients.

## 2. Molecular Aspects of Antibiotics Activity

There is an increased incidence of antibiotic resistance that is driven largely by inappropriate use of large volumes of antibiotics in animals, food and humans. The increased volume of antibiotics use results in increased selective pressure on bacteria which contributes to the development of resistance. There is a need to develop novel agents that work via different pathways to help overcome bacteria resistance. Recent studies have looked into novel agents of other pathways such as reactive oxygen species (ROS) and oxygen radicals, as an antimicrobial mechanism that may be effective in treating infections [17]. ROS have high antimicrobial activity against Gram-positive and Gram-negative bacteria, viruses and fungi; they also prevent and break down biofilm. ROS include superoxide anion (O^2−^), peroxide O_2_^−2^, hydrogen peroxide (H_2_O_2_), hydroxyl radicals (OH), and hydroxyl ions (OH^−^) [18]. ROS act as antimicrobials through a complex mechanism, i.e., hydrogen peroxide appears to directly elicit ROS’s antimicrobial action by its activity in thiol groups in enzymes and proteins, DNA and bacterial cell membrane. These compounds possess concentration-dependent activity and toxicity; and their half-life can be short. ROS can be delivered to the site of infection in various ways such as ROS gels allowing sustained continuous release of ROS to target sites [19]. Therefore, ROS can be used to treat local infections such as cavities, prosthetic devices and, by other delivery systems, to the respiratory and urinary epithelium. These functions make ROS highly suitable for chronic inflammatory conditions, where antibiotics are frequently overused and relatively ineffective, such as lung infections in patients with chronic lung diseases such as COPD. The first entirely novel antimicrobial agent to reach early clinical use employing ROS a mechanism has been developed for wound management [20]. ROS agents are also effective at preventing the formation of, and disrupting existing biofilm. These mechanisms can also have important application in respiratory conditions such as in patients undergoing mechanical ventilation [21].

Polysulfides are another substance that has recently been recognized for signaling ROS. Sulfites have been found to have a great role in the origin of life and are an important regulator and modulator of metabolism and signaling in all species including bacteria, and fungus [22]. Stepwise oxidation produces hydrogen persulfide radicals which can be oxidized to intermediate reactive sulfide species that work very similarly to ROS [23]. The “next frontier” of sulfide biology will be the understanding on these molecules and their effect in bacterial cell metabolism [24]. Therefore, the development of these novel antibacterial compounds using ROS could also have an important role in infection prevention and antimicrobial stewardship in chronic lung conditions.

COPD exacerbation is defined as an acute worsening of patients’ respiratory symptoms that results in additional therapy; these events can be precipitated by several factors; the most common cause is respiratory infections [25]. Compared to stable COPD, during ECOPD, a much larger percentage of patients have PPMs in addition to significantly higher concentrations of bacteria in the airways [26]. Treatment with appropriate antibiotics significantly decreases the bacterial burden by eradicating bacteria—reducing clinical failure and risk of progression to more severe infections, such as pneumonia [25,27].

While the increased airway inflammation present during ECOPD is reduced following antibiotic treatment, this resolution has been shown to be dependent on bacterial eradication [28]. Patients that have a relapse of their symptoms and/or required re-hospitalization could attribute this to persistent bacterial infection.

Among the major goals of COPD treatment in the current guidelines is the prevention of acute exacerbations [29]. Clinical studies have shown that long-term continuous or intermittent use of antibiotics has a beneficial effect of reducing exacerbation frequency and extending the time to the next exacerbation [30,31]. The mechanism underlying this improvement is unclear. The benefit of long-term antibiotic treatment could be related to changes in bacteria flora and changes in airway inflammation, but there are no clinical studies that support these hypotheses. Macrolides are known to have antibacterial and anti-inflammatory activity; recent data also suggested that they have antiviral activity and possibly disrupt biofilm formation in the airway. In 1987, Anthonisen, et al. [12] reported the results of a large-scale placebo-controlled trial designed to determine the efficacy of antibiotics in ECOPD. In this study, 173 COPD patients (mean FEV1(%) = 33%) were monitored for 3.5 years. Patients were classified based on their symptoms: Type 1 ECOPD patients had increased shortness of breath, increased sputum production, and change in sputum purulence and received any of the following antibiotics (amoxicillin, trimethoprim-sulfamethoxazole, co-trimoxazole, or doxycycline). In these patients, there was a significant improvement in symptoms as compared with placebo; there was no significant difference in the success rates between antibiotics and placebo in patients that had only one of these symptoms (called Type 3). Patients treated with antibiotics had a more rapid improvement in peak flow and a greater percentage of clinical success. In addition, the length of their illness was two days shorter for the antibiotic-treated group. The major limitation of this study was the lack of microbiology data; these investigators assumed that all antibiotics that they used for treating their patients were equivalent. It is important to point out that this study was conducted in the 1980s; since that time, we have seen significant changes in bacterial resistance and also in patients’ demographic characteristics. Allegra et al. [32] found a significant benefit using amoxicillin-clavulanate therapy compared with placebo in patients with moderate to severe disease. There was a significant success rate at day 5 in the antibiotics treated group (86% versus 50% in the placebo group, *p* < 0.01) and lower frequency of recurrent exacerbations. Another publication compared the efficacy of amoxicillin-clavulanate versus placebo in patients with mild and moderate COPD (patients with spirometry values FeV1 50–80%) that confirmed the findings of Allegra et al. [32]. These studies demonstrated the superiority of using antibiotics in these patients. Furthermore, the median time to the next exacerbation was also significantly prolonged in patients receiving antibiotics compared to placebo (233 days compared with 160 days, *p* < 0.05). Interestingly, this study demonstrated that sputum purulence was the most reliable marker of clinical failure in the placebo group [25]. A more recent study—a randomized, placebo-controlled trial—investigated the efficacy of doxycycline in addition to systemic corticosteroids in the treatment of hospitalized patients with ECOPD. This study showed that patients treated with doxycycline were not different to those in the placebo group regarding the primary end-point (clinical success at day 30) but showed superior results in some of the secondary end-points (clinical cure on day 10, microbiological success, open-label use of antibiotics and symptoms resolution). Although some of these outcomes are not clinically relevant, the antibiotic treatment was superior in patients with higher plasma levels of C-reactive protein [33]. The poor results observed with doxycycline at day 30 could be explained by the antibiotic bacteriological spectrum and local bacterial resistance patterns.

During an ECOPD, it has been suggested that antibiotics can reduce the burden of bacteria in the airway and, in some patients, can impact the progression of the event to more severe infections, such as pneumonia. A prospective, randomized, double-blind, placebo-controlled trial, evaluated the use of ofloxacin versus placebo in 90 patients with ECOPD who required mechanical ventilation; it showed that the antibiotic-treated group had a significantly lower in-hospital mortality rate (4% vs. 22%, *p* = 0.01) and reduced length of hospital stay (14.9 vs. 24.5 days, *p* = 0.01) compared with the placebo group. In addition, the ofloxacin-treated patients were less likely to develop pneumonia, especially during the first week of mechanical ventilation [27].

### Antibiotic Resistance

It has recently been recognized that antibiotic resistance is a major public-health problem worldwide, and international efforts are needed to counteract its emergence. Repeated and improper use of antibiotics is increasingly being recognized as the main cause of this emerging resistance [34]. Therefore, the identification of clinical characteristics that identify patients with ECOPD that can be safely treated without antibiotics is extremely important. In the case of mild to moderate ambulatory patients, the absence of sputum purulence and low values of C-reactive protein are associated with high rates of clinical cure without antibiotics [35]. Another study in hospitalized patients with ECOPD reported similar short- and long-term outcomes in patients with purulent sputum treated with antibiotics compared with patients with non-purulent sputum not treated with antibiotics. These data suggested that clinicians can use the presence or absence of changes in sputum color (purulence) as a way to limit the use of antibiotics; it is suggested that the use of antibiotics could be avoided in this latter group [36].

After the decision to initiate empirical antibiotic therapy, the choice of antibiotic must be considered. The reported relapse rates for patients with ECOPD range from 17% to 32%, and differ according to the antibiotics prescribed [37,38]. An international, multicenter study compared moxifloxacin to amoxicillin/clavulanic acid in patients with moderate to severe COPD (mean FEV1(%) = 39%) and clinical risk factors at 8 weeks post-therapy. There were no significant differences in the primary end-point of the study; however, moxifloxacin resulted in significantly lower clinical failure and higher bacteriological eradication in the sub-population of patients with bacterial pathogens isolated from sputum at inclusion [39]. These results suggest that, in confirmed bacterial ECOPD, the choice of antibiotic, particularly in severe patients, may result in different outcomes and justifies antibiotic selection based on patterns of antimicrobial resistance and the clinical characteristics of the patients.

## 3. Use of Antibiotics to Prevent Chronic Obstructive Pulmonary Disease Exacerbations

One of the unmet needs in the treatment of COPD is the prevention of COPD exacerbations in patients with recurrent bacterial infections. Long-term use of antibiotics has been suggested as a possible approach in these patients. In the last decade, several studies have been published showing the continuous long-term use of antibiotics in COPD patients [30,40,41,42,43] and one employing intermittent/pulsed treatment [23]. Suziki et al. [40] reported the first open-label study on erythromycin in the prevention of ECOPD. The investigators reported that the antibiotic-treated group showed a significant decrease in one or more exacerbations (11%) compared to the control group (56%) and less hospitalizations (*p* 0.007). Another study by Seemugal et al. [41] also showed that using erythromycin over a 12-month period led to a significant reduction in exacerbations but no differences in lung function changes or inflammatory markers. More recent publications showed significant reductions in inflammatory markers at 6 months with azithromycin [44] and erythromycin [43]. The most recent pivotal study evaluated the efficacy of daily azithromycin (250 mg/day) compared with placebo in a 12-month prospective trial in the prevention of COPD exacerbation [30]. These investigators reported that the use of antibiotics was associated with a 27% decrease in the frequency of exacerbation and significantly prolonged median time to an exacerbation. These investigators also reported that patients with moderate COPD, who were current smokers and had not been treated with long-acting bronchodilators were the most likely to benefit from the antibiotic therapy. More recently, Pomares et al. [42], in a retrospective study, showed significant reduction in exacerbations, hospitalizations and length of stay. The main concern related to the use of prophylactic antibiotics has been the development of bacterial resistance and the impact on the normal microbiota [37]. Another approach recently published by Sethi et al. [31] is on the intermittent use of antibiotics. In this study, the investigators use moxifloxacin given once daily for 5 days; the treatment was repeated every 8 weeks for a total of six courses of therapy. Although the study’s primary end-point was not met—a 25% reduction in exacerbations in the per-protocol population—in a post-hoc analysis, patients with moderate-severe COPD and with purulent or muco-purulent sputum at baseline showed a 45% decrease in exacerbations. It is also important to highlight that this study was not associated with increased bacterial resistance, but we do not know whether the investigators prolonged the clinical study to determine an association with the development of resistance [31]. Therefore, there is a need for long-term studies and also with different antibiotics to understand the efficacy of prophylactic therapy and the risk bacteria resistance.

The main issue that we will need to understand before we can recommend the “routine” use of antibiotics to prevent ECOPD is what is the impact on patients’ normal microbiota. For example, in the study by Albert et al. [30], patients that received azithromycin showed increased incidence of macrolide-resistant pathogens in nasopharyngeal swabs. Clearly, this intervention was affecting the individual’s normal microbiota [45].

## 4. Dosing Strategies of Antibiotics

There are no standard procedures that determine the dose and duration of antibiotic treatment in patients with ECOPD. The standard duration of antibiotic administration in ECOPD used to be 10 days. A shorter duration of therapy has very important advantages such as reduction of exposure that will result in decreased bacterial resistance and decreased side effects. Fallagas et al. [46] published a meta-analysis that included seven randomized controlled trials that demonstrated, in over 3083 patients, that short duration of antibiotics was as effective and safe as longer-therapy. Another study that included 21 double-blind studies showed that short-term antibiotics demonstrated clinical cure rates at both early and long-term follow-up; bacteriological response was also similar to that achieved with conventional therapy in patients with mild-moderate COPD exacerbations [47]. Therefore, these data demonstrate that short-term antibiotic use is associated with enhanced compliance, decreased resistance and costs. Furthermore, more recent studies demonstrated that a short course of antibiotics for 5 days using quinolones therapy was similar to long-term antibiotic treatment in patients with COPD exacerbation, as indicated by the clinical and bacteriological outcomes [48]. Similar findings were reported using high-dose quinolones with more rapid resolution of symptoms and faster recovery rates compared with traditional therapy with non-quinolones therapy [49].

## 5. Combination of Antibiotics and Systemic Corticosteroids

In severe COPD patients, the development of exacerbations is common in the use of both antibiotics and systemic corticosteroids. There is no clear data on whether antibiotics have additional benefits when given to patients that have also been treated with systemic corticosteroids. Sachs and colleagues [50] suggested that antibiotics did not provide additional clinical benefit when corticosteroids were given. These findings were irrespective of patients’ clinical characteristics such as sputum color or bacterial involvement. It is important to point out that this study had several limitations including a small sample size (*n* = 71), a mild population, and enrolled COPD and asthma patients. In a study by Daniels et al. [33], the lack of an effect with doxycycline in addition to systemic corticosteroids (the primary end-point being clinical success on day 30) may be related to the scarce antibacterial activity of doxycycline against pathogens such as *S. pneumoniae* and *H. influenza*; however, treatment with corticosteroids could help in patients with a more inflammatory response such as those with high C-reactive protein. More recent studies suggest that there are different phenotypes of COPD exacerbations, and systemic corticosteroids may be beneficial in those with predominant eosinophilic inflammation [51]. The different inflammatory profile of COPD exacerbations will need to be taken into consideration in the design of clinical trials examining the efficacy of antibiotics and/or corticosteroids in this disease. Today, we can only assess the host inflammatory response by non-specific markers such as C-reactive protein. It will be very interesting to design future clinical studies that take into consideration the host response in the randomization process to the presence or absence of antibiotics.

## 6. Measuring Effects and Outcomes

Clinical and microbiological end-points in clinical trials of antibiotic treatment of ECOPD are not well defined. Microbiological results depend on the production of a good quality sputum sample, which results in a positive sputum culture in only 20–50% of the patients. Clinical results are still based on the definition of Chow et al. [52]: “End-points are defined as cure (a complete resolution of signs and symptoms associated with the exacerbation) or improvement (a resolution or reduction of the symptoms and signs without new symptoms and signs associated with the exacerbation)”. “Clinical success is considered when either cure or improvement is observed”. “Failure is defined as incomplete resolution, persistence or worsening of symptoms that require a new course of antibiotics and/or oral corticosteroids or hospitalization”. Evaluation is usually performed at the end-of-therapy visit (days 9–14). This short time frame may not allow the identification of clinical relapses if they occur after initial improvement. Some antibiotics may decrease bacterial load sufficiently to produce an improvement in symptoms that can be perceived as a clinical success at the end of treatment, but when treatment is discontinued, the remaining microorganisms will increase in number and produce recurrent symptoms of exacerbation [53].

In patients with COPD, it is difficult to evaluate their symptoms both during stable conditions and exacerbations. In order to improve the recognition of patients’ symptoms, there is growing interest in the use of diary cards and standardized questionnaires to evaluate these conditions. The use of symptom-based diary cards may allow the quantification of the intensity and duration of patient symptoms over time and could be used to assess treatment outcomes [54,55,56]. There is a recent initiative, funded by regulatory agencies as well as pharmaceutical companies, called the Exacerbations of Chronic Pulmonary Disease Tool (EXACT) [57]. This is a new patient-reported outcome (PRO) diary that was developed to quantify patients’ daily symptoms before and after an exacerbation. The EXACT is a validated instrument that will aid in the quantification of the frequency, severity, and duration of exacerbations. It consists of 14 items that can be incorporated in the form of an e-diary, with scores ranging from 0 to 100 and higher scores indicating a more severe exacerbation. Some standardized quality-of-life questionnaires have been proven to be responsive to changes in health status during or after an exacerbation. The Saint George’s Respiratory Questionnaire (SGRQ) has been shown to be useful in monitoring recovery from ECOPD [58]. There is a derivative of the SGRQ that is called COPD Assessment Test (CAT), a short 8-item questionnaire that has been proven to provide a reliable score of patients’ symptoms both during stable conditions and exacerbation. The CAT score may also help to quantify the symptoms’ severity during exacerbations [59,60]. The generic European quality-of-life scale (EQ-5D) has been proven to be responsive to recovery from ECOPD [61,62] and is a good predictor of treatment failure [62]. The COPD Severity Score (COPDSS) is a severity scale developed by Eisner et al. [63] that is responsive to recovery from exacerbations and provides better predictive value for clinical success than that provided by the usual physiologic and clinical variables [51]. However, these quality-of-life or disease severity questionnaires have not been adequately tested in comparative clinical trials of therapies for ECOPD. In conclusion, the use of antibiotics in patients with COPD exacerbation should be limited to those patients with severe disease that have frequent exacerbations that required prior antibiotics use and or hospitalizations. It is important to point out that these patients should be treated with long-acting bronchodilators and anti-inflammatory therapy.

## 7. Clinical Guidelines

The current clinical guidelines of antibiotic treatment in ECOPD are based on the Anthonisen disease severity criteria [12] and recommend the use of antibiotics in those patients that have all three key symptoms (increased cough, purulence, and shortness of breath). In addition, antibiotics are also recommended in patients with severe ECOPD or hospitalized patients with only two of the three symptoms (increased purulence of sputum) and/or in patients that require invasive or non-invasive ventilation [13]. The Canadian Respiratory Society guidelines was the first publication to suggest the use of antibiotics based on the patient’s risk factors for poor outcome and correlated these findings with the most likely pathogens involved (Table 1) [64,65].

In general, COPD guidelines do not recommend the use of long-term antibiotics for the prevention of exacerbations. However, evidence of the efficacy of macrolides and, to a lesser extent, quinolones, has been accumulating over recent years. More recent guidelines have included, for the first time, a recommendation related to the long-term use of antibiotics in a specific subgroup of severe COPD patients that have chronic bronchitis, and or bronchiectasis [64,66]. These patients should have an early follow-up to evaluate side effects, such as deafness, and frequent sputum cultures to monitor bacteria resistance patterns. This treatment must be monitored closely for the possible development of side effects and/or changes in the patterns of bacterial resistance.

## 8. Future Developments of Antibiotics for COPD

Inhaled antibiotics have been developed to deliver lower doses that can obtain higher tissue concentration, maximizing pharmacodynamic parameters and minimizing systemic exposures. Inhaled antibiotics are widely used in the treatment of a number of respiratory tract infections, including cystic fibrosis (CF) [67] and bronchiectasis [68,69].

To date, there has been only one report investigating the use of inhaled antibiotics in patients with COPD. The study, conducted by Dal Negro et al. [70], reported the effect of nebulized tobramycin solution given for 14 days, twice daily, in patients with severe COPD. These investigators evaluated the clinical outcomes and inflammatory markers in patients that were colonized with multidrug-resistant *Pseudomonas aeruginosa*. This study demonstrated that two-week treatment with nebulized tobramycin resulted in a 42% decrease in the incidence of exacerbations compared with the prior 6 months and substantial reduction in pro-inflammatory markers. Ongoing and future trials using inhaled powder formulation of antibiotics (quinolones) will provide information on whether inhaled antibiotics are a useful therapeutic option in the prevention of ECOPD. Multiple clinical trials have been conducted (*clinicaltrials.gov*) on the use of inhaled antibiotics in patients with other chronic lung infections such as cystic fibrosis, and bronchiectasis; or as a prevention of infection in patients receiving mechanical ventilation; however, there are no studies that evaluate the use of inhaled antibiotics in COPD patients with exacerbations.

## 9. Conclusions

Chronic infections are associated with exacerbation in patients with COPD. Prevention and effective treatment of exacerbations are major objectives in the management of these patients. COPD exacerbations are associated with accelerated decline in lung function, worsening quality of life, increased morbidity, and mortality. Antibiotics are recommended for patients with severe COPD with an acute exacerbation that includes the presence of key clinical signs (increased sputum purulence and worsening shortness of breath). The use of antibiotics in COPD patients with an exacerbation and the presence of these symptoms is associated with clinical benefit, but treatment failure and relapse rates can also be high—mainly in cases of inadequate antibiotic therapy. Therefore, it is important to identify the patients at greatest risk of poor outcomes, since they are the patients who will likely derive the greatest benefits from early treatment with the most potent antibiotic therapy.

The long-term use of antibiotics remains controversial. While several studies showed beneficial effects—reducing frequency of exacerbations/hospitalizations and extending time to the next exacerbations—there are also concerns related to side effects and the development of bacterial resistance. Patients with frequent exacerbations and severe underlying disease will benefit from systemic antibiotic treatment during the exacerbation. In the future, more studies will also show that inhaled and/or nebulized routes will be effective.

## Figures and Tables

**Table 1:** Chronic obstructive pulmonary disease exacerbation risk classification based on patients’ clinical characteristics and most frequent microorganism.

Severity Classification	FEV1 (% Predicted)	Most Frequent Microorganisms
Mild to moderate COPD without risk factors	>50%	*H. influenzae*
		*M. catarrhalis*
		*S. pneumoniae*
		*C. pneumoniae*
		*M. pneumoniae*
Mild to moderate COPD with risk factors	>50%	*H. influenzae*
		*M. catarrhalis*
		PRSP
Severe COPD	30–50%	*H. influenzae*
		*M. catarrhalis*
		PRSP
		Enteric Gram negatives
Very severe COPD	<30%	*H. influenzae*
		PRSP
		Enteric Gram negatives
		*P. aeruginosa*

Risk factors include: age, use of prior antibiotics within the last 4–6 weeks, prior exacerbations. FEV1: forced expiratory volume in one second. PRSP: penicillin-resistant *S. pneumoniae*. Modified from ref. [64,65].
